# Nonlinear association between systemic inflammation response index and mortality in adult cardiac surgery–associated acute kidney injury: A retrospective Cohort study based on the MIMIC-IV database

**DOI:** 10.1097/MD.0000000000049168

**Published:** 2026-06-26

**Authors:** Tong Tan, Alimujiang Awaker, Yongqiang Lai

**Affiliations:** a Department of Cardiovascular Surgery Center, Beijing Anzhen Hospital, Beijing Institute of Heart, Lung and Blood Vascular Diseases, Capital Medical University, Beijing, China.

**Keywords:** cardiac surgery–associated acute kidney injury, immune homeostasis, inflammation, MIMIC-IV, mortality, Systemic Inflammation Response Index

## Abstract

Systemic inflammation plays a pivotal role in the pathogenesis and prognosis of cardiac surgery–associated acute kidney injury (CSA-AKI). The Systemic Inflammation Response Index (SIRI) has emerged as a novel biomarker reflecting immune inflammatory balance. This study aimed to explore the association between SIRI and in-hospital mortality among patients with CSA-AKI. Data of CSA-AKI patients were extracted from the MIMIC-IV 3.1 database. SIRI was calculated and divided into 3 tertiles. Restricted cubic spline (RCS) models were used to assess nonlinear associations between SIRI and mortality. Multivariate logistic regression models were constructed to evaluate the independent relationship between SIRI and outcomes after adjusting for potential confounders. A total of 5394 patients with CSA-AKI were included. Patients in the medium SIRI tertile (1.27–3.06) demonstrated the lowest mortality (1.3%) compared to the low (2.0%) and high (3.1%) tertiles. RCS analysis revealed a significant U-shaped association between SIRI levels and in-hospital mortality risk (*P* for nonlinearity < .001). Below the inflection point (SIRI = 1.6899), mortality risk tended to decrease with increasing SIRI, while above this threshold, each 1-unit increase in SIRI was significantly associated with a 10% higher risk of in-hospital mortality (OR 1.10, 95% CI 1.03–1.16, *P* = .001) after adjustment. SIRI demonstrated a U-shaped association with mortality among patients with CSA-AKI, indicating that both immune suppression and excessive inflammation are detrimental. SIRI may serve as a simple, readily available biomarker to reflect immune homeostasis and assist in postoperative risk stratification. Further prospective studies are warranted to validate these findings.

## 1. Introduction

Acute kidney injury (AKI) is a frequent and severe complication following cardiac surgery, affecting 20 to 40% of patients and substantially increasing the risk of morbidity and mortality.^[1,2]^ Despite advances in perioperative care, early identification of patients at high risk for adverse outcomes after cardiac surgery–associated AKI (CSA-AKI) continues to pose a major clinical challenge. In recent years, the Systemic Inflammation Response Index (SIRI) has emerged as a novel composite biomarker that integrates the host immune and inflammatory responses and has gained attention as a potential prognostic tool in critically ill patients. Elevated SIRI levels have been associated with adverse outcomes in a variety of cardiovascular conditions. For example, high SIRI values independently predicted major adverse cardiovascular events in patients with acute coronary syndrome and chronic kidney disease.^[3]^ Similarly, higher SIRI was shown to predict long-term mortality in sepsis-associated AKI patients.^[4]^ However, its prognostic value in CSA-AKI patients has not been previously explored. The correlation between SIRI criteria and clinical outcomes does not always follow a simple linear pattern. Studies show it may follow a nonlinear pattern, where both low and high values denote immune dysregulation and are associated with increased mortality risk.^[5–7]^ Our study aimed to investigate the association between SIRI and in-hospital mortality in patients with CSA-AKI using data from the Medical Information Mart for Intensive Care IV (MIMIC-IV) database.

## 2. Materials and methods

### 2.1. Data source

Data for this retrospective cohort study were obtained from the MIMIC-IV database. MIMIC-IV is a publicly available, large-scale critical care database developed collaboratively by the Laboratory for Computational Physiology at the Massachusetts Institute of Technology (MIT), Beth Israel Deaconess Medical Center (BIDMC), and Philips Healthcare. It integrates de-identified electronic health records from BIDMC, a major academic tertiary hospital in Boston, Massachusetts. MIMIC-IV (version 3.1) includes 94,458 unique intensive care unit (ICU) stays recorded between 2008 and 2022.^[8,9]^ It contains detailed clinical information, including demographics, laboratory test results, vital signs, medications, surgical procedures, diagnoses, and survival outcomes. Further details are available on the PhysioNet platform (https://physionet.org/content/mimiciv/3.1/). Access to the MIMIC-IV database was approved by the institutional review boards of MIT and BIDMC, and all data are fully de-identified. Therefore, the requirement for individual informed consent was waived. Access to the database is granted only to researchers who have completed the National Institutes of Health training course “Protecting Human Research Participants” and have obtained authorization to use the data. The use of this database for the current study was authorized through the MIT data use agreement (Record ID: 39150437). Data extraction was performed using Structured Query Language implemented in PostgreSQL (version 15.15), and database management and query execution were supported by Navicat Premium (version 16).

### 2.2. Study population

Patients recorded in the MIMIC-IV 3.1 database who underwent cardiac surgery during their hospital stay were considered for inclusion in this study. Cardiac surgical procedures were identified according to ICD-9 and ICD-10 procedure codes, encompassing aortic, mitral, or tricuspid valve operations, coronary artery bypass grafting (CABG), thoracic aortic interventions, and combined cardiac procedures. Eligible participants met the following criteria: age 18 years or older; underwent at least 1 of the above cardiac surgical procedures during the index hospitalization; were admitted to ICU postoperatively; and developed AKI after surgery. In accordance with the Kidney Disease: Improving Global Outcomes (KDIGO) criteria,^[10]^ AKI was identified if any of the following conditions were met: an increase in serum creatinine (Scr) of ≥ 0.3 mg/dL within 48 hours; an increase in Scr to ≥ 1.5 times the baseline value within 7 days; or a urine output below 0.5 mL/kg/h for at least 6 hours. Exclusion criteria included: non-first ICU admissions during the index hospitalization; missing laboratory parameters necessary for SIRI calculation; and extreme SIRI values outside the 1^st^ and 99^th^ percentiles of the distribution, which were trimmed to reduce the influence of potential measurement or recording errors.

### 2.3. Exposure and outcome

SIRI was defined as the main exposure variable in this study. For each included patient, SIRI was calculated using the complete blood count before and closest to the time of postoperative AKI onset. Specifically, the SIRI value was derived from the neutrophil, monocyte, and lymphocyte counts according to the formula: SIRI (×10^[9]^/L) = neutrophil count (×10^[9]^/L) × monocyte count (×10^[9]^/L)/ lymphocyte count (×10^[9]^/L).^[11]^ Based on the distribution of SIRI, patients were categorized into 3 groups for subsequent comparative and survival analyses.

The primary outcome of this study was in-hospital mortality, defined as death occurring during the index hospitalization after cardiac surgery. The secondary outcome was 28-day all-cause mortality, defined as death from any cause within 28 days after surgery.

### 2.4. Statistical analysis

Continuous variables were tested for normality using skewness and kurtosis values. Normally distributed variables were presented as mean ± standard deviation and compared across SIRI tertiles using 1-way analysis of variance, while non-normally distributed variables were expressed as median (interquartile range) and compared using the Kruskal–Wallis test. Categorical variables were summarized as counts and percentages and compared with the Chi-square test or Fisher exact test as appropriate.

Survival outcomes were analyzed using the Kaplan–Meier method. The time-to-event was defined as the number of days from the date of cardiac surgery (t_0_) to either death or censoring at 28 days. Patients who survived beyond 28 days were censored at that time point. Survival curves were compared across SIRI tertiles using the log-rank test, and 95% confidence intervals (CI) were computed based on the log–log transformation. To explore the potential nonlinear relationship between SIRI and in-hospital mortality, a restricted cubic spline (RCS) model was fitted using logistic regression with 4 knots. The analysis was performed using the rms package in R. Multivariable logistic regression models were constructed to examine the association between SIRI and in-hospital mortality. To further characterize the dose–response pattern, a piecewise logistic regression model was built using the nadir value of SIRI as the knot, estimating separate slopes for SIRI below and above this threshold. Subgroup analyses were performed to evaluate the consistency of associations across predefined strata, including age (<60 or ≥ 60 years), sex, BMI (<30 or ≥ 30 kg/m^2^), emergency admission (yes or no), combination cardiac surgery (yes or no), and SOFA score (below or above the median).

## 3. Results

### 3.1. Baseline characteristics

**Figure 1.**
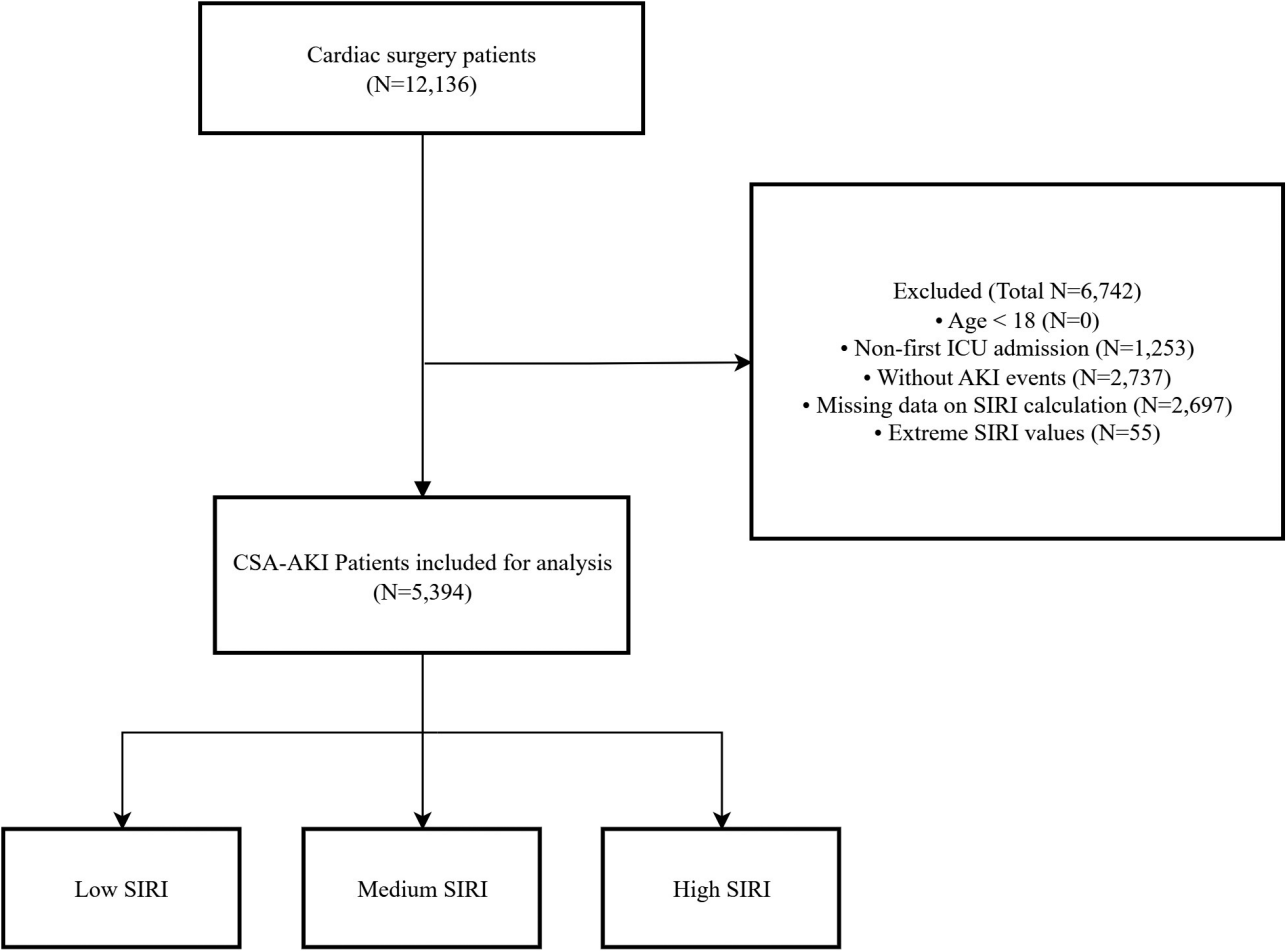
The flowchart of patients’ selection. SIRI = Systemic Inflammation Response Index.

Patients received valves, CABG, thoracic aortic interventions, or combined cardiac procedures were involved. After applying the exclusion criteria, 6742 patients were excluded: 1253 patients with non–first ICU admissions, 2737 patients without AKI events, 2697 patients with missing data required for SIRI calculation, and 55 patients whose SIRI values exceeded biologically plausible ranges. Finally, a total of 5394 CSA-AKI patients were included in the final analysis. According to tertiles of SIRI, patients were categorized into 3 groups: low SIRI (≤1.26), medium SIRI (1.27–3.06), and high SIRI (>3.06), with 1798 patients in each group (Fig. 1). The overall mean age of the cohort was 68.6 ± 11.3 years, and 28.1% were female. As shown in Table 1, baseline demographic and clinical characteristics differed significantly among the 3 SIRI groups. Patients in the high SIRI group were more likely to be male, had a higher BMI, and were more frequently White or of other ethnicities compared with those in the low SIRI group (*P* < .001 for all). The distribution of surgery types also varied, with the high SIRI group having a greater proportion of valve and aortic surgeries, while CABG procedures were more common in the low SIRI group (*P* < .001). With respect to comorbidities, higher SIRI levels were associated with an increased prevalence of myocardial infarction, congestive heart failure, and diabetes mellitus (all *P* < .01).

**Table 1:** Baseline characteristics of CSA-AKI patients.

Variables	Overall (N = 5394)	Low SIRI (N = 1798)	Medium SIRI (N = 1798)	High SIRI (N = 1798)	*P*-value
**Age, years**	68.6 ± 11.3	69.1 ± 11.3	68.3 ± 10.9	68.4 ± 11.5	.051
**Female, n (%**)	1517 (28.1%)	664 (36.9%)	463 (25.8%)	390 (21.7%)	<.001
**BMI, kg/m^2^**	28.7 (25.4, 32.9)	28.0 (24.9, 31.8)	29.0 (25.6, 33.1)	29.2 (25.7, 33.6)	<.001
**Ethnicity, n (%**)					<.001
** Asian**	98 (1.8%)	43 (2.4%)	30 (1.7%)	25 (1.4%)	
** Black**	220 (4.1%)	125 (7.0%)	58 (3.2%)	37 (2.1%)	
** White**	3905 (72.4%)	1215 (67.6%)	1330 (74.0%)	1360 (75.6%)	
** Others**	1171 (21.7%)	415 (23.1%)	380 (21.1%)	376 (20.9%)	
**Emergency admission, n (%**)	381 (7.1%)	140 (7.8%)	115 (6.4%)	126 (7.0%)	.264
**Surgery type, n (%**)					<.001
** CABG**	2730 (50.6%)	954 (53.1%)	941 (52.3%)	835 (46.4%)	
** Valve surgery**	1045 (19.4%)	293 (16.3%)	324 (18.0%)	428 (23.8%)	
** Thoracic aorta**	192 (3.6%)	70 (3.9%)	49 (2.7%)	73 (4.1%)	
** Combination**	1427 (26.5%)	481 (26.8%)	484 (26.9%)	462 (25.7%)	
**Co-morbidities**					
** Myocardial infarct, n (%**)	1708 (31.7%)	546 (30.4%)	541 (30.1%)	621 (34.5%)	.006
** Congestive heart failure, n (%**)	1730 (32.1%)	492 (27.4%)	558 (31.0%)	680 (37.8%)	<.001
** Peripheral vascular disease, n (%**)	947 (17.6%)	327 (18.2%)	308 (17.1%)	312 (17.4%)	.68
** Cerebrovascular disease, n (%**)	591 (11.0%)	218 (12.1%)	181 (10.1%)	192 (10.7%)	.128
** Chronic pulmonary disease, n (%**)	1132 (21.0%)	367 (20.4%)	385 (21.4%)	380 (21.1%)	.749
** Rheumatic disease, n (%**)	184 (3.4%)	71 (3.9%)	48 (2.7%)	65 (3.6%)	.09
** Diabetes, n (%**)	1992 (36.9%)	721 (40.1%)	653 (36.3%)	618 (34.4%)	.001
**Laboratory data**					
** WBC, K/ul**	12.1 (8.9, 15.6)	8.6 (6.4, 11.4)	12.1 (9.8, 14.6)	15.7 (12.8, 19.3)	<.001
** Neutrophils, K/ul**	9.3 (6.6, 12.3)	6.2 (4.4, 8.1)	9.2 (7.5, 11.3)	12.8 (10.3, 15.6)	<.001
** Monocytes, K/ul**	0.4 (0.2, 0.6)	0.2 (0.1, 0.3)	0.4 (0.3, 0.6)	0.7 (0.6, 1.0)	<.001
** Lymphocytes, K/ul**	1.8 (1.3, 2.5)	1.9 (1.4, 2.6)	2.0 (1.4, 2.6)	1.7 (1.2, 2.4)	<.001
** Baseline creatinine, mg/dL**	1.0 (0.8, 1.2)	1.0 (0.8, 1.2)	1.0 (0.8, 1.2)	1.0 (0.8, 1.3)	.057
** Lactate, mmol/L**	2.0 (1.5, 2.6)	1.9 (1.5, 2.6)	1.9 (1.5, 2.5)	2.0 (1.5, 2.8)	<.001
**Charlson comorbidity index**	4.6 ± 2.3	4.7 ± 2.3	4.5 ± 2.3	4.7 ± 2.4	<.001
**SOFA**	5.5 ± 2.8	5.5 ± 2.8	5.3 ± 2.7	5.8 ± 2.8	<.001
**SAPSII**	37.0 (31.0, 44.0)	36.0 (31.0, 43.0)	36.0 (30.0, 43.0)	37.0 (31.0, 45.0)	<.001
**LODS**	4.0 (3.0, 6.0)	4.0 (3.0, 6.0)	4.0 (3.0, 6.0)	5.0 (3.0, 6.0)	<.001
**RRT, n (%**)	268 (5.0%)	97 (5.4%)	64 (3.6%)	107 (6.0%)	.003
**In-hospital mortality, n (%**)	115 (2.1%)	36 (2.0%)	24 (1.3%)	55 (3.1%)	.001
**28-day mortality, n (%**)	106 (2.0%)	36 (2.0%)	26 (1.4%)	44 (2.4%)	.096

BMI = body mass index, CABG = coronary artery bypass grafting, CSA-AKI = cardiac surgery–associated acute kidney injury, LODS = Logistic Organ Dysfunction System score, RRT = renal replacement therapy, SAPS II = simplified acute physiology score II, SIRI = Systemic Inflammation Response Index, SOFA = Sequential Organ Failure Assessment, WBC = white blood cell.

### 3.2. Association between SIRI and mortality outcomes

**Figure 2.**
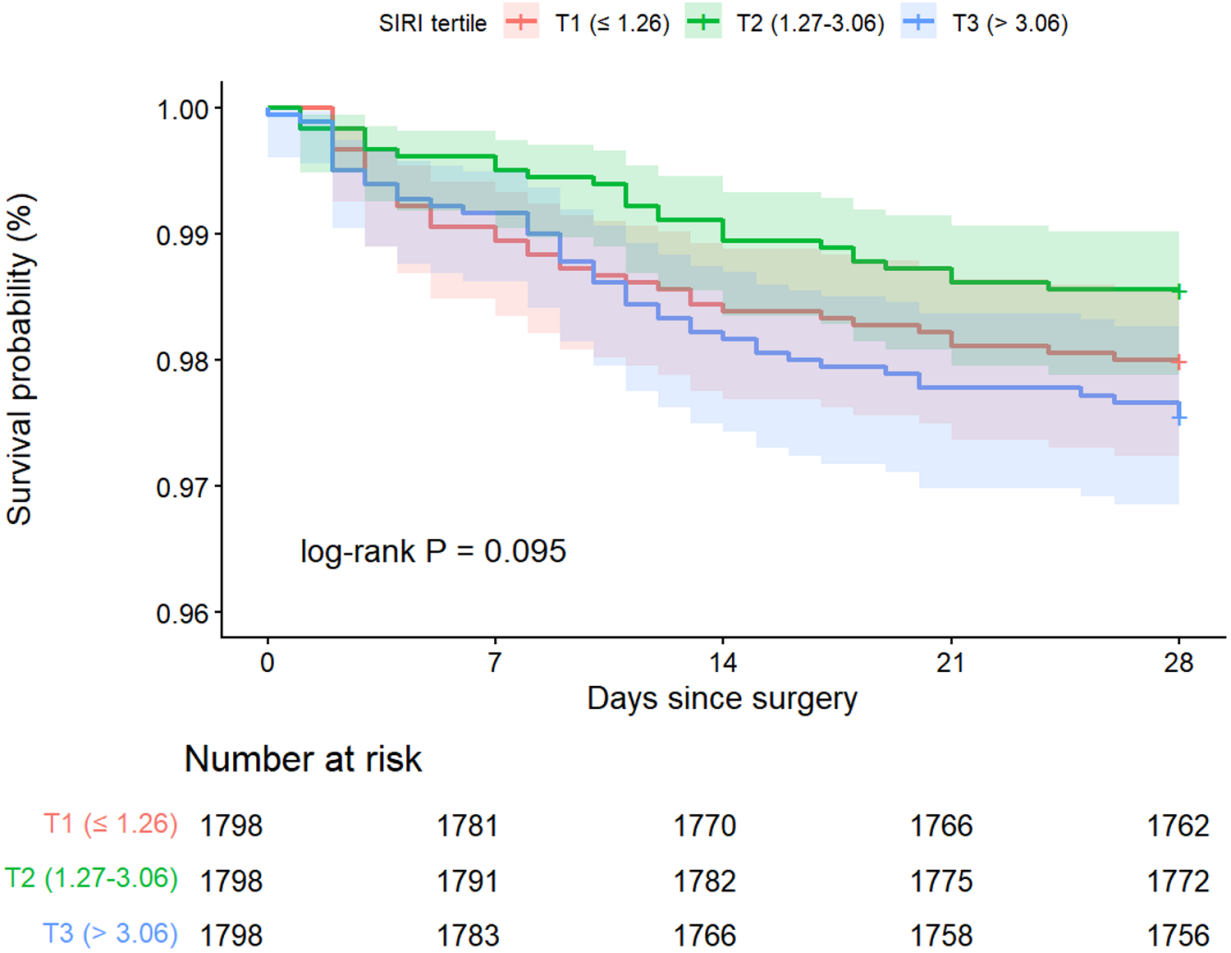
Kaplan–Meier survival curves showing 28-day all-cause mortality among CSA-AKI patients stratified by SIRI tertiles. CSA-AKI = cardiac surgery–associated acute kidney injury, SIRI = Systemic Inflammation Response Index.

The overall in-hospital mortality rate was 2.1%. The in-hospital mortality rate differed significantly among the 3 SIRI groups (2.0%, 1.3%, and 3.1% for low, medium, and high groups, respectively; *P* = .001). The 28-day mortality rate was 2.0%. Note that it was lower than the in-hospital mortality rate, as some patients remained hospitalized for more than 30 days and were therefore not captured within the 28-day window. The 28-day Kaplan–Meier survival curves are shown in Figure 2. Patients in the medium SIRI group (98.6%) exhibited the highest survival probability, whereas both the low SIRI (98.0%) and the high SIRI (97.6%) groups showed slightly lower survival rates. Although the difference in 28-day mortality did not reach statistical significance (log-rank *P* = .095), it suggests a potential U-shaped relationship between SIRI levels and short-term mortality risk.

To quantify this nonlinear relationship, a piecewise logistic regression model was constructed using the nadir value (SIRI = 1.6899) as the reference point (Table S1). Among patients with SIRI values ≥ 1.6899, each 1-unit increase in SIRI was associated with a 10% higher risk of in-hospital mortality after full adjustment (OR 1.10, 95% CI 1.03–1.16). In contrast, lower SIRI values were associated with reduced mortality risk in partially adjusted models; however, this association was attenuated after further adjustment for markers of disease severity.

**Figure 3.**
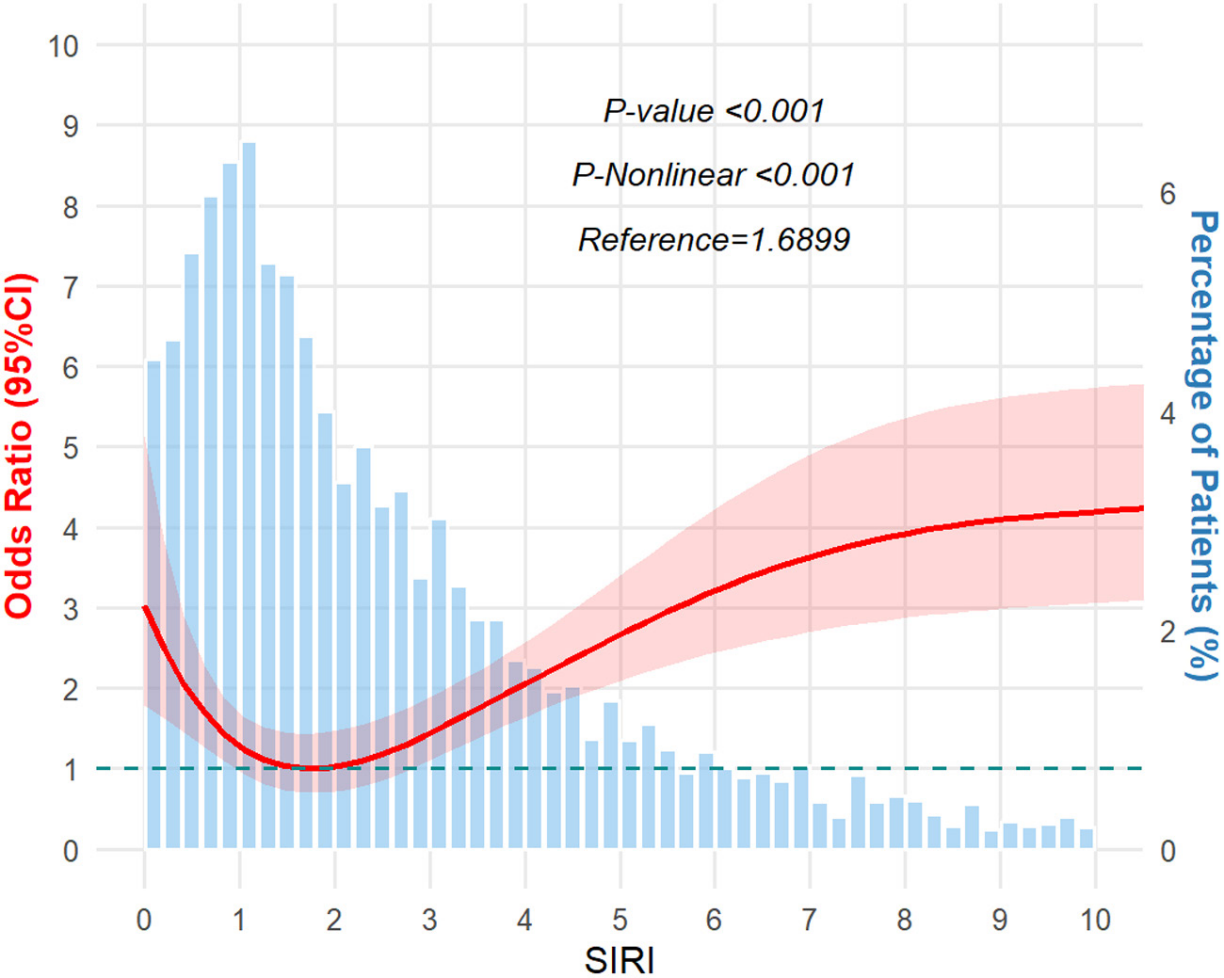
The restricted cubic spline curve for the association of SIRI with the in-hospital mortality of CSA-AKI patients. SIRI = Systemic Inflammation Response Index, CSA-AKI = cardiac surgery–associated acute kidney injury.

Restricted cubic spline (RCS) analysis was performed (Fig. 3) and demonstrated a significant nonlinear association between SIRI levels and mortality risk (*P* for overall association < .001; *P* for nonlinearity < .001). The risk of in-hospital death decreased sharply with increasing SIRI at very low levels, reached its lowest point around a SIRI value of 1.6899 (OR = 1), and then increased progressively with further elevation of SIRI, forming a U-shaped relationship. The association of SIRI with in-hospital mortality was further investigated by employing 3 multivariate logistic regression models (Models 1, 2, and 3) (Table 2). Across all models, the medium SIRI group consistently demonstrated the lowest odds of in-hospital mortality (Model 3: OR = 0.77, 95% CI: 0.41–1.43). Based on these models, a piecewise logistic regression model was applied, using a SIRI value of 1.6899 as the inflection point (Table S1). Below this threshold (SIRI < 1.6899), the risk of in-hospital mortality tended to decrease with increasing SIRI, although the association was not statistically significant in the fully adjusted model (Model 3: OR = 0.75, 95% CI: 0.48–1.19, *P* = .207). In contrast, above the inflection point (SIRI ≥ 1.6899), higher SIRI levels were significantly associated with an increased risk of in-hospital death (Model 3: OR = 1.10, 95% CI: 1.03–1.16, *P* = .001).

**Table 2:** Association between Systemic Inflammation Response Index (SIRI) tertiles and in-hospital mortality among postoperative acute kidney injury after cardiac surgery.

SIRI	Model 1 OR (95%CI)	Model 2 OR (95%CI)	Model 3 OR (95%CI)
T1	Ref.	Ref.	Ref.
T2	0.71 (0.42, 1.19)	0.68 (0.38, 1.18)	0.77 (0.41, 1.43)
T3	1.68 (1.09, 2.61)	1.26 (0.78, 2.03)	1.23 (0.73, 2.10)
*P for trend*	0.012	0.277	0.389

Model 1 was adjusted for age, sex, and ethnicity.

Model 2 was further adjusted for body mass index, surgery type, emergency admission, myocardial infarction, congestive heart failure, and diabetes.

Model 3 was additionally adjusted for lactate level and Sequential Organ Failure Assessment (SOFA) score.

CI = confidence interval, OR = odds ratio, SIRI = Systemic Inflammation Response Index.

### 3.3. Subgroup analyses

Subgroup analyses were conducted to examine whether the association between the systemic inflammatory response index (SIRI) and in-hospital mortality varied across clinically relevant subgroups, including sex, age, body mass index, emergency admission status, surgical complexity, and baseline organ dysfunction as assessed by the SOFA score (Figure S1). No significant interactions were observed between SIRI and any subgroup variable for either segment of the piecewise model (all *P* for interaction > .05), indicating that the association between SIRI and mortality was generally consistent across subgroups.

## 4. Discussion

SIRI has emerged as a promising marker of systemic inflammation and has shown prognostic value across multiple clinical conditions. Elevated SIRI levels have been linked to worse outcomes in cardiovascular disease, sepsis, and chronic kidney disease.^[12,13]^ For instance, Qu et al confirmed that higher SIRI was associated with an increased risk of major adverse cardiovascular events after myocardial infarction or percutaneous coronary intervention.^[14]^ However, patients undergoing cardiac surgery represent a particularly distinctive population characterized by intense perioperative immune activation and ischemia–reperfusion injury, both of which contribute critically to AKI and postoperative mortality. The prognostic behavior of composite inflammatory indices such as SIRI in this setting has not been elucidated. To our knowledge, this is the first study to demonstrate a U-shaped association between SIRI and in-hospital mortality specifically within the CSA-AKI population, revealing that both low and high SIRI levels are linked to increased risk, while moderate levels are protective. This nonlinear relationship was consistently observed in RCS and piecewise regression analyses and remained evident after rigorous multivariable adjustment. These results suggest that SIRI may serve as a valuable prognostic biomarker in CSA-AKI patients and reflect underlying pathophysiological mechanisms linking systemic inflammation and clinical outcomes.

Compared to single inflammatory ratios such as the neutrophil-to-lymphocyte ratio or platelet-to-lymphocyte ratio, SIRI offers a more comprehensive reflection of systemic immune dynamics by integrating neutrophilic, monocytic, and lymphocytic components. This multidimensional approach captures both hyperinflammatory and immunosuppressive states, thereby serving as a sensitive marker of immune equilibrium. The nadir identified in our cohort closely aligns with thresholds reported in cardiovascular populations, where SIRI values between approximately 1.5 and 2.0 have been associated with the lowest event rates.^[15]^ A high SIRI indicates overactivation of innate immunity together with suppression of adaptive responses. It reflects an imbalance where neutrophils and monocytes dominate, amplifying systemic inflammation, while lymphocyte depletion weakens the regulatory control that normally restrains this process. This “double-hit” pattern represents a breakdown of immune homeostasis in the perioperative setting.^[16]^ During cardiac surgery, cardiopulmonary bypass (CPB) acts as a priming pro-inflammatory and hemolytic stimulus. The extracorporeal circuit subjects circulating erythrocytes to unphysiological shear stress and mechanical trauma, precipitating intravascular hemolysis and the subsequent systemic liberation of cell-free hemoglobin (CFH) and free heme. These circulating heme species catalyze the formation of reactive oxygen species and scavenge nitric oxide, thereby amplifying oxidative stress, promoting endothelial dysfunction, and impairing microvascular vasodilation.^[17,18]^ CFH/heme also serve as damage-associated molecular patterns that engage innate immune signaling and promote neutrophil recruitment and activation. Once activated, neutrophils release neutrophil extracellular traps (NETs)—DNA–protein complexes designed to trap pathogens but which, in sterile postoperative inflammation, adhere to the renal microvascular endothelium and propagate injury. Experimental and clinical studies have demonstrated that NET deposition within renal capillaries increases endothelial permeability, promotes microvascular congestion, and accelerates medullary hypoxia, key features of CSA-AKI.^[19–21]^ Complementary to neutrophil injury pathways, the activation of monocytes and macrophages triggers a secondary wave of injury via the secretion of pro-inflammatory cytokines such as TNF-α, IL-6, and IL-1β.^[22]^ These mediators directly compromise renal autoregulation and provoke afferent arteriolar vasoconstriction, thereby intensifying the ischemia–reperfusion injury inherent to aortic cross-clamping. This synergistic effect ultimately drives cortical perfusion deficits and facilitates renal tubular apoptosis.

A low SIRI in the context of CSA-AKI may suggest a maladaptive immunological state characterized by a compensatory antiinflammatory response and immune exhaustion. In critical illness, the initial pro-inflammatory surge is physiologically counterbalanced by a compensatory antiinflammatory response syndrome, wherein profound down-regulation of innate and adaptive immune effectors occurs to limit collateral damage from unchecked inflammation.^[23]^ However, extended CPB or trauma induced by cardiac surgery is also associated with subsequent leukocyte dysfunction and reduced antigen-presenting capacity, suggesting a shift toward immunoparalysis. Furthermore, this state of immune hyporeactivity may predispose patients to a higher risk of delayed postoperative infections. After cardiac surgery, quantitative and qualitative changes in cellular immunity, including depressed T-cell function and altered neutrophil/lymphocyte dynamics, persist into the postoperative period, aligning with an adaptive yet pathologically blunted inflammatory response. Moreover, the resolution of AKI similarly requires an organized sequence of immune events. Early pro-inflammatory monocytes and M1 macrophages are crucial for clearing necrotic debris and initiating repair, whereas subsequent polarization toward M2 macrophages supports resolution and tissue regeneration.^[24]^ A persistently low SIRI may thus signal a blunted immunological transition; without a sufficient early inflammatory response to drive debris clearance, the subsequent recruitment of monocytes remains too sparse to facilitate the M2-mediated repair necessary for tubular recovery. This mechanistic gap in the “injury-to-repair” continuum could help explain why low SIRI patients experience elevated mortality. It is not from acute collateral organ damage as in high SIRI states, but from failed recovery and persistent organ dysfunction.

From a clinical standpoint, the U-shaped association observed implies that both hyperinflammatory and immunoparalytic trajectories confer excess mortality in CSA-AKI. SIRI, as an inexpensive and readily available biomarker, may serve as a practical adjunct for perioperative immune monitoring, enabling early identification of patients at risk for maladaptive immune responses. Those with persistently elevated SIRI might benefit from anti-cytokine or antioxidant strategies, whereas those with low SIRI may require immunostimulatory or infection surveillance approaches.

This study was conducted on a large cohort of patients undergoing cardiac surgery, which enhances the generalizability of our findings across diverse clinical settings. However, several limitations should be acknowledged. First, this was a retrospective, single-center study, and potential selection bias cannot be fully excluded. Second, SIRI was measured at a single postoperative time point; thus, dynamic changes in inflammatory status throughout the perioperative course were not captured. Finally, the study did not explore whether SIRI could continue to serve as a predictive or monitoring biomarker during the therapeutic course of CSA-AKI. Future prospective studies incorporating serial SIRI measurements and clinical interventions are warranted to determine whether SIRI-guided immune monitoring can improve outcomes in this high risk population.

## 5. Conclusion

In summary, this study identifies a significant U-shaped association between SIRI and in-hospital mortality among patients with CSA-AKI, indicating that both excessively low and high systemic inflammatory states are linked to adverse outcomes. SIRI may help clinicians assess postoperative immune status and refine risk stratification in CSA-AKI. These results advocate for the integration of SIRI into clinical monitoring to facilitate personalized immunomodulatory strategies, though future prospective trials are warranted.

## Acknowledgments

We gratefully acknowledge the dedicated work of the teams involved in the development and maintenance of the MIMIC-IV database.

## Author contributions

**Conceptualization:** Tong Tan, Yongqiang Lai.

**Data curation:** Tong Tan.

**Formal analysis:** Tong Tan, Alimujiang Awaker.

**Methodology:** Tong Tan.

**Writing – original draft:** Tong Tan.

**Writing – review & editing:** Tong Tan, Alimujiang Awaker, Yongqiang Lai.




