# Supplementary material for: Nonlinear association between systemic inflammation response index and mortality in adult cardiac surgery–associated acute kidney injury: A retrospective Cohort study based on the MIMIC-IV database
Source: Medicine (Baltimore). 2026 Jun 26;105(26):e49168. doi: 10.1097/MD.0000000000049168 (PMC13313715; doi:10.1097/MD.0000000000049168)
Supplement: Supplementary file 1 [file medi-105-e49168-s001.docx]

Supplemental Table 1. Piecewise associations between SIRI and in-hospital mortality.

| **Model** | **SIRI segment** | **OR (95% CI)** | **P value** |
| --- | --- | --- | --- |
| Model 1 | SIRI < 1.6899 | 0.71 (0.48–1.06) | 0.085 |
|  | SIRI ≥ 1.6899 | 1.15 (1.10–1.20) | <0.001 |
| Model 2 | SIRI < 1.6899 | 0.63 (0.42–0.97) | 0.031 |
|  | SIRI ≥ 1.6899 | 1.12 (1.07–1.18) | <0.001 |
| Model 3 | SIRI < 1.6899 | 0.75 (0.48–1.19) | 0.207 |
|  | SIRI ≥ 1.6899 | 1.10 (1.03–1.16) | 0.001 |

SIRI = Systemic Inflammation Response Index; OR = odds ratio; CI = confidence interval.
