# Supplementary figures and images for: Nonlinear association between systemic inflammation response index and mortality in adult cardiac surgery–associated acute kidney injury: A retrospective Cohort study based on the MIMIC-IV database
Source: Medicine (Baltimore). 2026 Jun 26;105(26):e49168. doi: 10.1097/MD.0000000000049168 (PMC13313715; doi:10.1097/MD.0000000000049168)

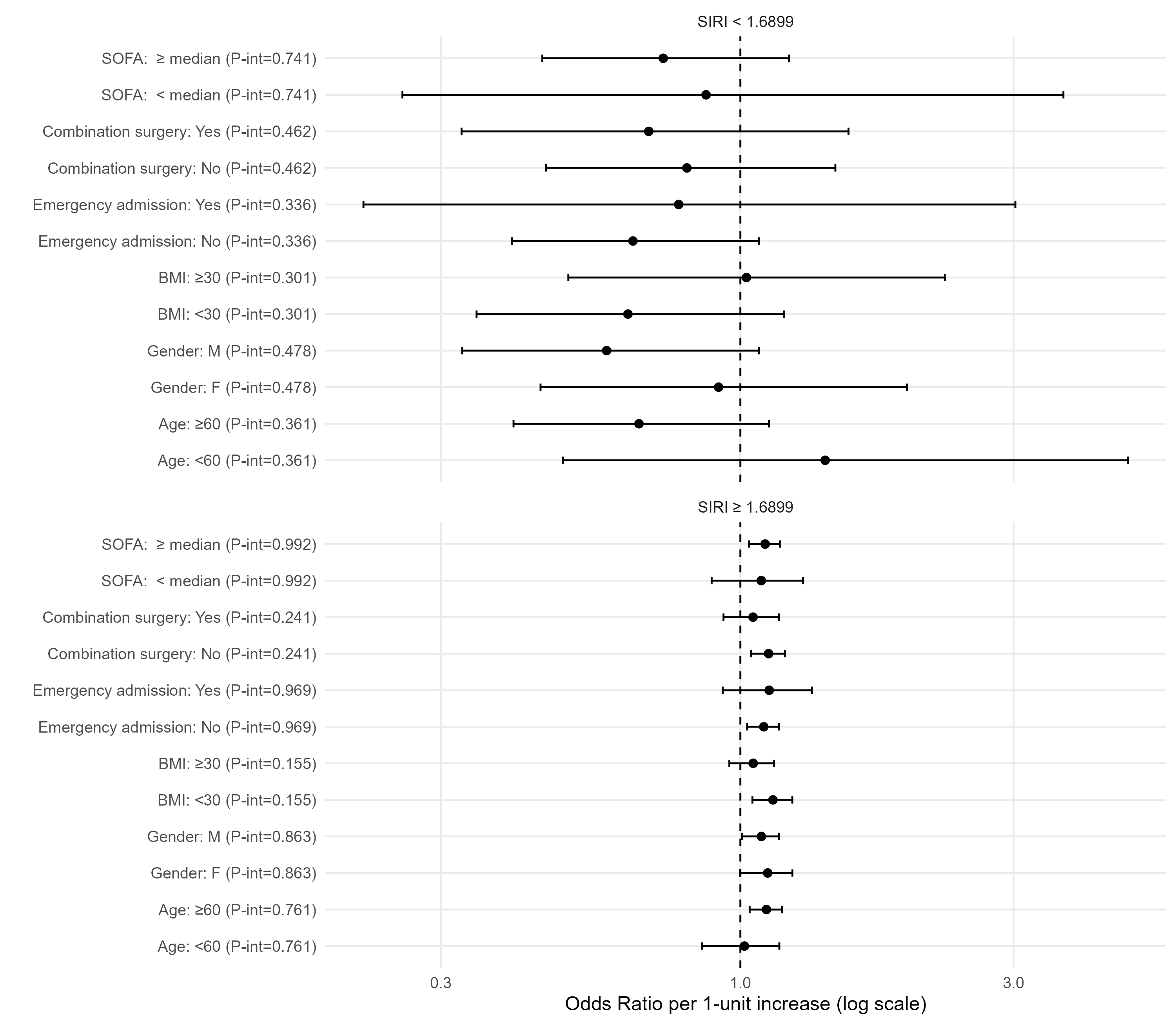

Supplement: Supplementary file 2 [file medi-105-e49168-s002.tiff]
